# The Impact of Annealing Methods on the Encapsulating Structure and Storage-Stability of Freeze-Dried Pellets of Probiotic Bacteria

**DOI:** 10.1007/s11095-024-03751-w

**Published:** 2024-07-29

**Authors:** Shuai Bai Palmkron, Björn Bergenståhl, Stephen Hall, Sebastian Håkansson, Marie Wahlgren, Emanuel Larsson, Anna Millqvist Fureby

**Affiliations:** 1 Department of Process and Life Science Engineering, Division of Food and Pharma Lund University, 221 00 Lund, Sweden; 2 Division of Applied Microbiology, Department of Chemistry, Lund University, 221 00 Lund, Sweden; 3 BioGaia AB, 241 38 Eslöv, Sweden; 4 Chemical Process and Pharmaceutical Development, RISE Research Institutes of Sweden, Stockholm, Sweden; 5 Department of Experimental Medical Science, Lund University, 221 00 Lund, Sweden; 6 LUNARC, Lund University, Box 118, 221 00 Lund, Sweden; 7 Division of Solid Mechanics, Department of Construction Sciences, Lund University, 22100 Lund, Sweden

**Keywords:** annealing, collapse, encapsulated bacteria, freeze-drying, oxygen barrier, pellets, relaxation, shelf-life, structural analysis, tomography

## Abstract

**Objective:**

This paper investigates the critical role of material thickness in freeze-dried pellets for enhancing the storage stability of encapsulated bacteria. Freeze dried material of varying thicknesses obtained from different annealing durations is quantified using Scanning Electron Microscopy (SEM) and X-ray microtomography (μCT), the material thickness is then correlated to the storage stability of the encapsulated cells.

**Methods:**

A formulation comprising of sucrose, maltodextrin, and probiotic cells is quenched in liquid nitrogen to form pellets. The pellets undergo different durations of annealing before undergoing freeze-drying. The material thickness is quantified using SEM and μCT. Storage stability in both oxygen-rich and oxygen-poor environments is evaluated by measuring CFU counts and correlated with the pellet structure.

**Results:**

The varying annealing protocols produce a range of material thicknesses, with more extensive annealing resulting in thicker materials. Storage stability exhibits a positive correlation with material thickness, indicating improved stability with thicker materials. Non-annealed pellets exhibit structural irregularities and inconsistent storage stability, highlighting the impracticality of avoiding annealing in the freeze-drying process.

**Conclusions:**

Extensive annealing not only enhances the storage stability of probiotic products but also provides greater control over the freeze-drying process, ensuring homogeneous and reproducible products. This study underscores the importance of material thickness in freeze-dried pellets for optimizing storage stability for probiotic formulations, and emphasize the necessity of annealing as a critical step in freeze-drying quenched pellets to achieve desired structural and stability outcomes.

**Supplementary Information:**

The online version contains supplementary material available at 10.1007/s11095-024-03751-w.

## Introduction

Pellets are one of the most used formats of industrial freeze-drying for probiotics. However, most scientific studies on the freeze-drying of probiotics are carried out using vials or trays (1–6). There are important differences, between the freeze-drying of pellets and vials, in freeze-drying performance, as well as in the structure of the freeze-dried material (7). One principal aspect is that the quenching of pellets in liquid nitrogen results in small ice crystals with a fine structure and non-equilibrium freezing that results in a freeze concentrate with low Tg (8), while the comparably slow cooling inside a freezer or freeze-dryer results in a much coarser structure (7). The different freezing methods also exert very different kinds of stress on the bacteria (9). The structure of ice crystals in the frozen pellet affects the final structure of the freeze-dried product. One way of modifying the ice structure in a sample is to expose the sample to a temperature between the glass transition temperature and the freezing point, a so-called annealing process. Annealing allows larger ice crystals to grow while the smaller crystals shrink and disappear, the process is usually termed Ostwald ripening. Factors that increase the rate of annealing are short distances between ice crystals, large surface area from which the water molecule can diffuse, and high Laplace pressure (10). A sample with small ice crystals has a short distance between each other, a large surface, and high Laplace pressure. The Ostwald ripening is therefore especially efficient for quenched pellets with small ice crystals. However, the effects of annealing are more limited in vial freezing as the ice crystals after crystallization already are comparatively large, and thus the kinetics of the process are slow (7, 11).

Numerous studies have investigated the impact of annealing on the drying rate, physical properties and cake structure of freeze-dried protein and pharmaceutical formulations(12–16). However, there are only few studies focusing on the effects of annealing on probiotics. In a study by Ekdawi-Sever et al. involving pellets containing probiotics, it was observed that short annealing times resulted in smaller pores and thinner freeze-dried material, and structural collapse in less annealed samples. Their findings indicated that annealing adversely affected storage stability, which they attributed to low residual moisture, no conclusions were drawn regarding the structural differences between annealed and non-annealed pellets(11).

The heat transfer in vial freeze-drying primarily originates from the heat conduction from the shelf through the bottom of the vial (about 60–70%) (17), while the heat transfer for pellets is solely through radiation. A pellet formulation also has a significant increase in surface area per volume exposed to the vacuum, which facilitates mass transfer. The lack of conductive heat transfer allows for a significant increase in the shelf temperature, while the temperature at the sublimation front remains controlled by the pressure (which prevents collapse during drying) and results in more efficient drying. After freeze-drying, the cells are encapsulated in a protective material often consisting of an amorphous non-reducing sugar, that protects the cell membrane integrity (18), and a longer oligomer/polymer such as maltodextrin to increase the stability during storage. The hypothesis for this paper is that the thickness of the encapsulating material has an impact on protecting the cells from oxidative stress (8). This is based on that sugars in a glassy state provide a good barrier towards oxygen and act as a buffering material for the humidity (19, 20). A thicker protective material is also expected to be less sensitive to structural movements during storage. Thus, we expect to observe that thicker material structures provide better protection of the cells and provide a slower loss of viability during storage. The material thickness was measured using X-ray microtomography (µCT) and scanning electron microscope (SEM).

To evaluate the storage stability of bacteria in formulations that have different structures, three different protocols were designed;1) anneal the sample under “aggressive and fast” conditions, 2) anneal under mild and slow conditions, and 3) compared with a reference condition without annealing. To compare the impact of oxygen on storage stability, the pellets were stored in plastic canisters permeable to oxygen and containing a desiccant and in aluminum vacuum bags that act as an oxygen barrier.

## Materials and Methods

### Fermentation

The fermentation of *Limosilactobacillus reuteri* DSM 17938 was performed in two 1L Multifors-bioreactors (Infors HT, Switzerland) and monitored using eve® software (Infors HT, Switzerland). The bioreactors were cooled down to 10 °C and inoculated with 10 ml preculture provided by BioGaia (Eslöv, Sweden), the temperature was then ramped up to 37 °C. MRS broth (GranuCult, Millipore) was used as a growth medium, and the pH was maintained stable at 5.5 using 3 M KOH. The base consumption was used to monitor the growth of the cells and the program was stopped when the pH became stable (after approx. 9 h). The fermentation was performed without aeration and a stirrer speed of 250 RPM.

### Formulation and Production of Pellets

The content of the two reactors was pooled after fermentation and the number of viable cells was counted using a flow cytometer CytoFLEX (Beckman Coulter, US). It resulted in a final concentration of 1.9·10^9^ cells/ml. The number of viable cells was used to obtain the desired viable cell concentration in the formulations. Further, the sample was centrifuged and washed with phosphate buffer saline solution. A formulation was created with a total dry matter of 20% (w/w) consisting of 2/3 sucrose (for molecular biology, Sigma-Aldrich) and 1/3 maltodextrin 9 (hydrolyzed potato starch, dextrose equivalent 8–10, from Roquette, France). The formulation was mixed with the cells to obtain a final concentration of 2·10^11^ cells/ml. The suspension was then pumped using a peristaltic pump at 20 ml/min and quenched by dripping it into liquid nitrogen. The pellets were spherical and ranged from 4–5 mm in diameter. The time between mixing the formulation with the cells and the freezing was kept short (less than 15 min). The obtained pellets were directly transferred and stored in a -80 °C freezer. For the annealing process, the pellets were divided into three equal parts; whereas one underwent an annealing process at -20 ± 0.5 °C in a freezer for a week (to emulate an industrial storage situation), another part underwent a quick annealing process at -9 °C for 2 h, and the last part remained at -80 °C and did not undergo any annealing process (referred to as non-annealed). After the different annealing processes, all the pellets were stored in a -80 °C freezer until freeze-drying.

### Freeze-Drying of Pellets

Both batches of prepared frozen pellets were loaded into aluminum cups and suspended inside an Epsilon 2-6D LSC plus Freeze dryer from Martin Christ equipped with Pirani, piezo, and capacitance pressure sensors (Germany) that had been pre-cooled to -30 °C. The loading of the pellets into the freeze dryer from the -80° C freezer took approximately 10–15 min per batch and as a result, Batch 1 of the pellet was subjected to -30 °C for a longer time compared to Batch 2. Once the pellets were loaded, the pressure inside the chamber was decreased to 30 Pa. When the desired pressure was reached, the shelf temperature was increased to 20 °C at a rate of 4.5 °C/min. There was no distinction between the primary and secondary steps. Instead, the entire drying process was performed under the same conditions. The end of the drying process was determined to be at 18 h after the time point when the Pirani and capacitance sensors showed identical pressure readings.

### Determination of Glass Transition Temperature, Water Content, and Water Activity

The glass transition temperature of maximally freeze concentrated solution (Tg’) of 2/3 sucrose, and 1/3 maltodextrin with a total dry weight of 20% (w/w) was analyzed using differential scanning calorimeter DSC (Mettler Toledo, Switzerland). Triplicates of 10 mg material were sealed in 40 µl aluminum pans. The samples were cooled to -70 °C at a rate of 10 °C/min, followed by a 5-min equilibration and a subsequent heating step to 30 °C at the same rate. The Tg' were determined using STARe Software according to ISO standard (ISO 11357–2:1999).

The water content of the dried pellets was analyzed directly after freeze-drying. The measurements were performed using Thermogravimetric analysis (TGA) with a TA Q500 (TA Instruments, New Castle, DE, USA). 2–3 crushed pellets from each batch were loaded onto an open platinum pan, and the temperature was then increased from 25 °C to 200 °C at a heating rate of 10 °C/min. The weight decrease was recorded from 25 °C to 125 °C to avoid the inclusion of water released due to Maillard and caramelization reactions.

Water activity was measured at the same time point as the storage stability using a Rotronic HC-2 Water activity probe (PST, Ely, UK), measurements below 0.01 are denoted as < 0.01 as values below this level are regarded inaccurate. All measurements are done with intact pellets without any signs of visible collapse or cracks.

### Freeze-Drying Survival and Storage Stability

Freeze-drying survival was examined directly after harvesting the pellets from the freeze-dryer. 1 g of pellets were first dissolved in MRS broth and then diluted and plated using an automatic plater Easy spiral dilute (Interscience, France) on MRS agar and incubated at 37 °C for 48 h. The CFU was counted using a scan 500 colony counter (Interscience, France) and compared to the initial cell concentration (defined using a flow cytometer). An accelerated stability study was conducted at 37 °C and 75% RH. All pellets (including pellets with cracks and visual collapse) were divided into equal parts approximately 1 g per sample and stored in plastic canisters with a drying agent (silica gel) and in vacuum-sealed aluminum bags. The storage stability was determined by analyzing CFU at each time point with freeze-drying survival as the reference point. Five time points were investigated for pellets stored in canisters, where a new canister was evaluated for each time point. The time points investigated were 7, 14, 28, and 56 days. Vacuum-sealed bags were investigated at 28 and 56 days.

The half-life of the bacteria was estimated from the viability data assuming exponential decay over the observation time:

1 $$C\left({t}_{end}\right)=C\left({t}_{start}\right){e}^{-\frac{{t}_{2}-{t}_{1}}{\lambda \left({t}_{m}\right)}}$$

2 $$\lambda ({t}_{m})=\frac{{t}_{end}-{t}_{start}}{\text{ln}(C\left({t}_{start}\right))-\text{ln}(C\left({t}_{end}\right))}$$

3 $${t}_{1/2}=\frac{\text{ln}(2)}{\lambda \left({t}_{m}\right)}$$

where *C(t)* is the number of viable bacteria (per gram) at time *t*, *t*_*start*_ and *t*_*end*_ are the starting and ending times of the observation period, and *t*_*m*_ is the average of the observation period. λ*(t)* is the decay constant. The decay constant is assumed to depend on factors such as water activity, structural changes, diffusive equilibration processes etc.

### Scanning *Electron* Microscopy

One intact pellet without any visible sign of collapse or cracks from each preparation protocol and batch was selected for scanning electron microscopy (SEM) analysis. The pellets were carefully divided into halves using a razor blade. The divided samples were then fixed to a sample holder and sputtered with a layer of gold–palladium (in a 60:40 ratio) with an expected film thickness of 9 nm. The pellets were investigated using a SEM microscope (JEOL JSM-6700F, Tokyo, Japan). 50x, 200x, and 800 × magnification were used to study the pellet’s structure. Additionally, a higher magnification of 2000 × was used to investigate the bacteria embedded in the material.

### X-ray Microtomography

The X-ray microtomography (µCT) was performed on Zeiss Xradia Versa 520 (Zeiss, Heidelberg, Germany). One annealed pellet with no sign of macroscopic collapse or cracks was selected from each batch and condition. The selected samples were cut into ¼ wedges using a razor blade. The samples were then securely wedged into 200 μl pipette tips. The tips were sealed with parafilm to prevent moisture absorption. An overview scan of 1920 μm x 1920 μm x 1920 μm and a local zoomed-in scan of 614 μm x 614 μm x 614 μm were conducted on the top half of the wedge. The scanning parameters used are presented in Table I. The image analysis follows the previously established methodologies addressed in (7, 8).

**Table I:** Parameters used for the µCT

	Overview scan (2 μm pixel size)	Local scan (0.64 μm pixel size)
Source voltage	80 kV	80 kV
Source current	87uA	87uA
Source power	7W	7W
Exposure time per projection	0.3 s	3.0 s
Number of projections	1601	3201
Zoom (Lens)	4x	20x
Source to sample distance	9.03 mm	9.03 mm
Sample to detector distance	21.4 mm	9.81 mm
Effective pixel size	2.00 μm	0.64 μm

The greyscale image obtained from the tomography was treated with a 3D median filter of 4 × 4x4 pixels followed by a standard grey-level thresholding. Further quantitative image analysis was performed using the Python software package PoreSpy (porespy.org) to calculate the material thickness (referred to as local thickness) of the freeze-dried material. In PoreSpy, the local thickness procedure inscribes a number of 'blobs' with a diameter corresponding to the material thickness in the material. The counts of the blobs were presented as a frequency distribution, indicating the number of blobs at each diameter. However, the objective is to understand the distribution of the material thickness rather than a number of blobs. To accomplish this, the number distribution was transformed into a volume-weighted distribution.

The volume-weighted average of the material thickness is calculated from the volume-weighted average of the measuring ‘blobs’.

4 $${d}_{vol}=\frac{{\int }_{0}^{V}d dV}{{\int }_{0}^{V} dV}\approx \frac{\sum_{j=1}^{J}{d}_{j} {V}_{j}}{\sum_{j=1}^{J}{V}_{j}}$$

where *d*_*vol*_ is the material thickness, *V* is the volume of all material in the analyzed 3D volume. *d*_*j*_ is the diameter of the blobs. The volume-weighted average thickness is obtained from the blobs in each class *j* counted over *J* classes. ${V}_{j}$ is the total volume of all particles in class *j*.

## Results

### Appearance after Freeze-Drying

**Fig. 1:**
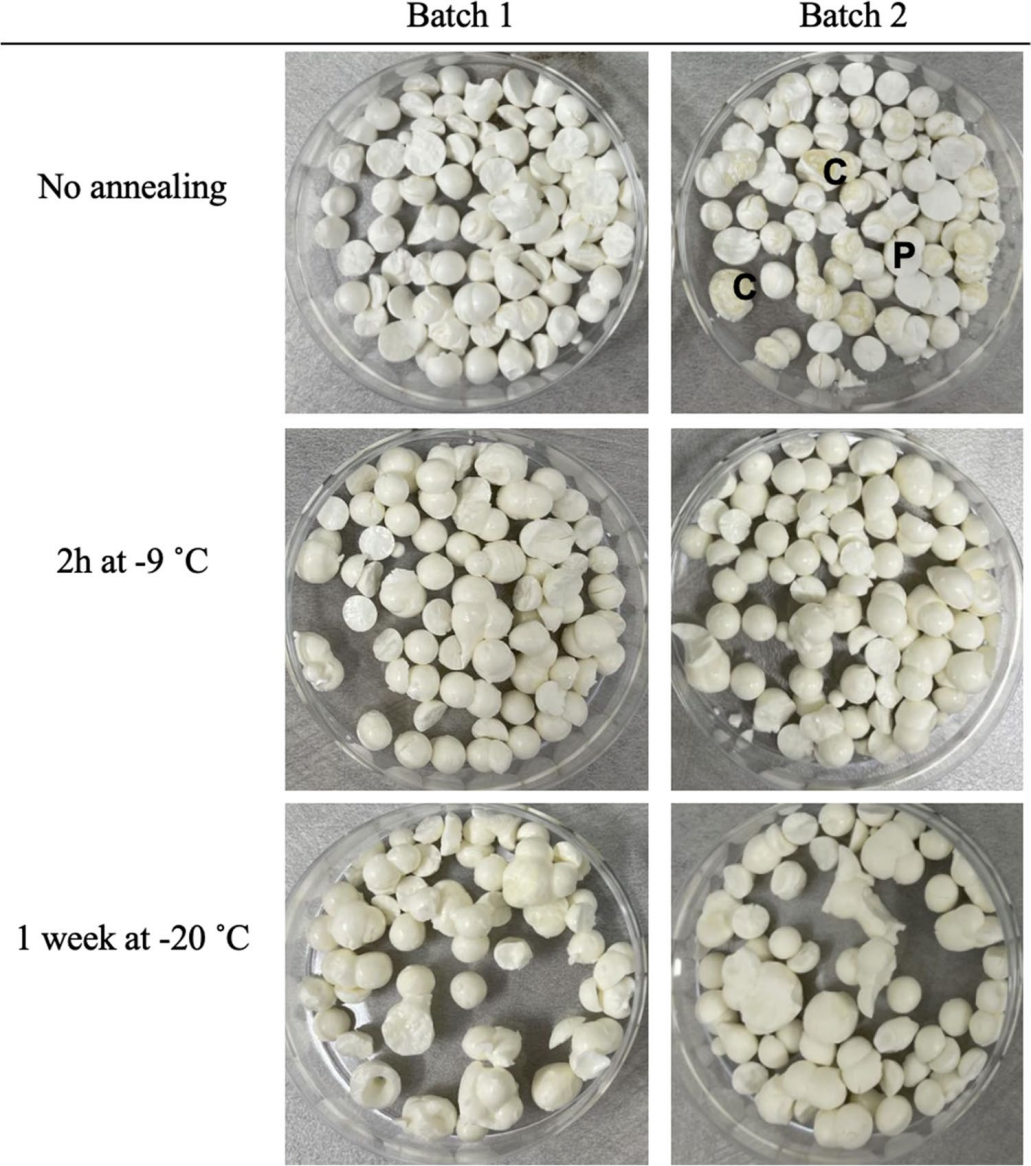
Appearance of freeze-dried pellets. Notice the difference between the non-annealed batches, where Batch 2 has significantly more collapse indicated by observable bubbles and cavities and a more uneven and shinier surface. C indicates visually collapsed, and P indicates visually intact pellets, respectively

The appearance of the pellets is shown in Fig. 1. There were no noticeable changes in volume before and after freeze-drying. The annealed pellets displayed a more yellowish surface and were less fragile compared to the non-annealed. There was no sign of macroscopic collapse or pellet-to-pellet variation within the protocols except for the non-annealed pellets in Batch 2. Around 1/3 of the pellets in this batch had visual signs of collapse, where some of the pellets showed observable bubbles and cavities and a more uneven and shinier surface.

### Water Content, Glass Transition Temperature and Water Activity

The water content and activity of the pellets are presented in Table II*.* The results show that the water content of the dried pellets was 3.5 to 4.0%. There was a larger deviation for the non-annealed samples, while the annealed samples had a more consistent water content.

**Table II:** The Water Content and Water Activity (during storage) for the Individual Batches

Sample		Water content Day1 (%)	Water activity Day 1	Water activity Day 28	Water activity Day 56
Non-annealed (Canister)	Batch 1	3.3	0.029	< 0.01	< 0.01
	Batch 2	3.9	0.03	< 0.01	< 0.01
2 h at -9˚C (Canister)	Batch 1	3.6	0.027	< 0.01	< 0.01
	Batch 2	3.4	0.028	< 0.01	< 0.01
1 week at -20˚C (Canister)	Batch 1	3.9	0.031	< 0.01	< 0.01
	Batch 2	4.0	0.031	< 0.01	< 0.01
Non-annealed (Vacuum-bag)	Batch 1	3.3	0.029	0.026	0.034
	Batch 2	3.9	0.03	0.056	0.081
2 h at -9˚C (Vacuum-bag)	Batch 1	3.6	0.027	0.037	0.042
	Batch 2	3.4	0.028	0.021	0.036
1 week at -20˚C (Vacuum-bag)	Batch 1	3.9	0.031	0.037	0.042
	Batch 2	4.0	0.031	0.033	0.046

The onset of the glass transition temperature, Tg’ for the formulation 20% (w/w) consisting of 2/3 sucrose and 1/3 maltodextrin was determined to be -25˚C and the onset of the melting temperature, Tm, at -9˚C. Due to the interference from the cells in the formulation, the glass transition temperature of the freeze-dried pellets could not be experimentally determined.

**Fig. 2:**
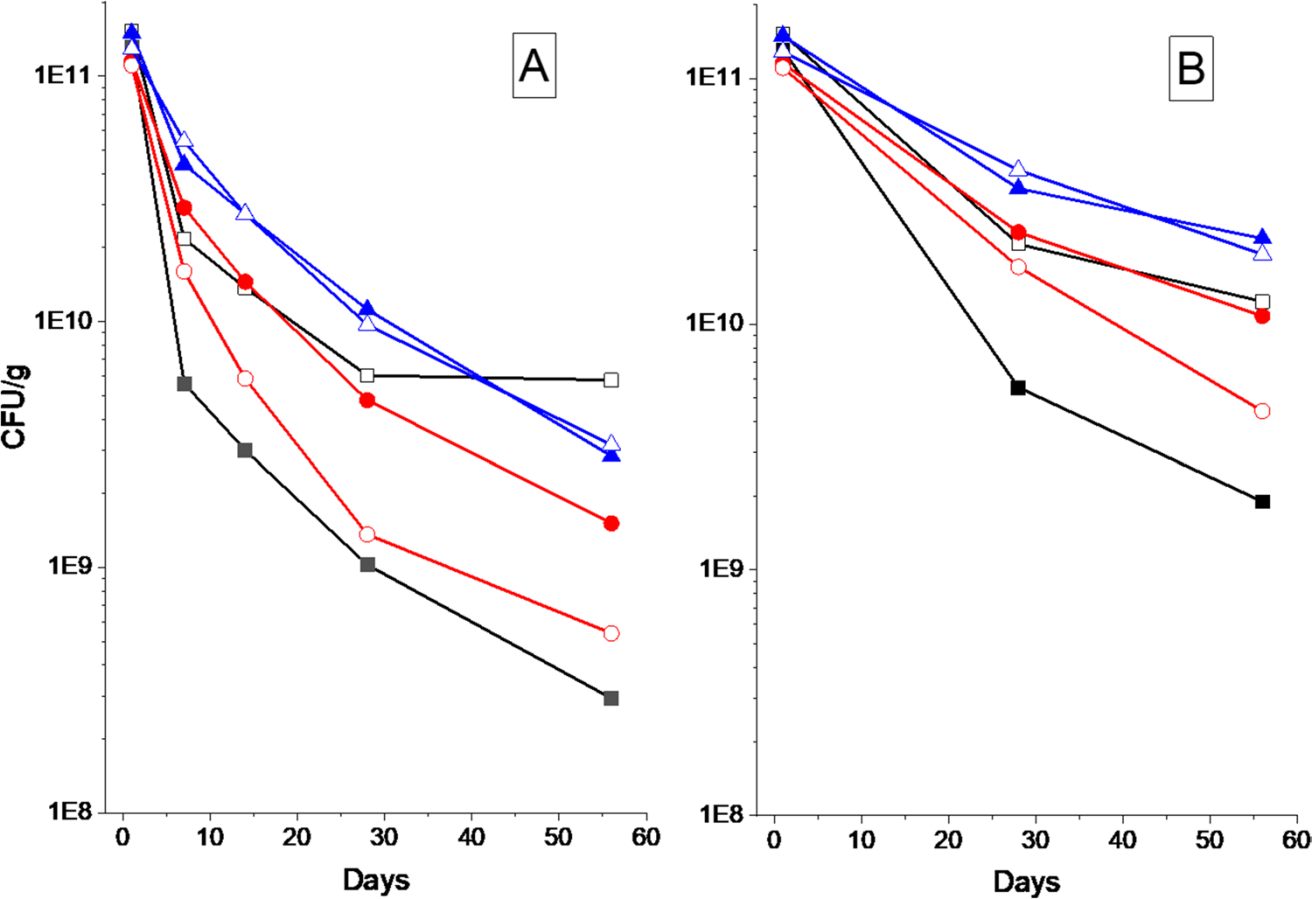
Survival of freeze-dried Limosilactobacillus reuteri in pellets after storage at 37°C for 56 days. (Batch 1 ■, Batch2 □) Non-annealed, (Batch 1●, Batch 2 ○) annealed for 2h at -9°C, and (Batch 1 ∆, Batch 2 ▲) annealed for 1 week at -20°C. A) samples stored in a canister with desiccant, and B) samples stored in aluminium vacuum bags

The water activity for all samples directly after freeze-drying was low at around 0.03. For the subsequent time points, the pellets stored in canisters with a drying agent had a water activity below 0.01 and were thus, too low to be measured accurately. The water activity for pellets stored in vacuum-sealed bags remained in a measurable range and increased over time. The water activity is presented in Table II. It can be observed that the non-annealed pellets from Batch 2 had a notably higher water activity compared to pellets in Batch 1 after storage for 28 and 56 days in a vacuum bag (Fig. 2).

### Freeze-Drying Survival and Storage-Stability

The freeze-drying survival and half-life during storage is shown for both batches and all annealing procedures in Table III. The freeze-drying survival were highest for the non-annealed samples and the samples annealed for one week at -20 ˚C at around 80% of the initial viable cell concentration. The freeze-drying survival was lower for in pellets that had undergone a rapid annealing for 2 h at -9 ˚C resulting in a survival of 65%.

**Table III:** Summary of Freeze-Drying Survival, the Half-Life during Storage, and Average Material Thickness based on Tomography. Half-Life is Calculated According to Eq. 1–3 Using Data from Fig. 2

Sample		Freeze-drying survival (%)	Half-life Time 0 to 1 month [days]	Half-life 1 month to 2 months [days]	Material thickness, Mode value (Fig. 5) [µm]
Non-annealed (Canister)	Batch 1	75	4.0	6.4	0.5^a^
	Batch 2	87	6.0	11.9	N/A^b^
2 h at -9˚C (Canister)	Batch 1	66	6.1	8.9	2.3
	Batch 2	64	4.5	7.3	2
1 week at -20˚C (Canister)	Batch 1	85	7.5	9.8	3
	Batch 2	73	7.5	10.4	3
Non-annealed (Vacuum-bag)	Batch 1	75	6.1	9.2	0.5^a^
	Batch 2	87	9.8	15.5	N/A^b^
2 h at -9˚C (Vacuum-bag)	Batch 1	66	12.3	16.4	2.3
	Batch 2	64	10.4	12.1	2.0
1 week at -20˚C (Vacuum-bag)	Batch 1	85	13.5	20.4	3.0
	Batch 2	73	17.4	20.4	3.0

^a^ Estimated from SEM (Fig. 3)

^b^ Heterogeneous sample

### Structure of the Freeze-Dried Pellets

#### SEM

**Fig. 3:**
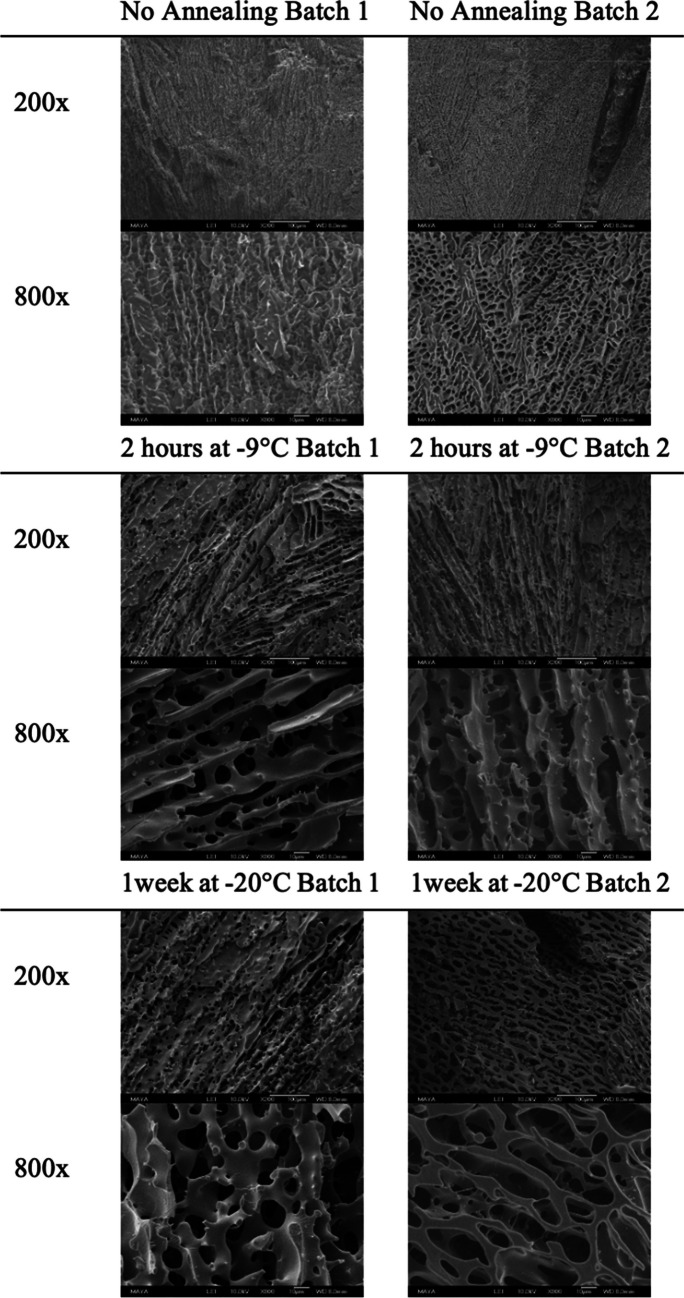
SEM images of the pellets’ inner structure taken at 200 × and 800x. One intact pellet without any signs of collapse or crack from each protocol and batch was chosen

SEM images were taken from the middle of the pellet at 200 × and 800 × magnification (Fig. 3). An overview scan of the pellets at 50 × magnification and a highly magnified image to detect the cells at 2000 × are presented in the Supplementary Material.

The non-annealed samples have a dense fine structure with small pores around 5 μm. The pores are aligned in a pattern pointing towards the centre of the pellet. The material thickness is estimated from the SEM images as being around 0.5 µm. There are parts of the non-annealed pellets, where microscopic collapse has occurred, and these parts have a much larger pore size and thicker material. The micro-collapse is inhomogeneous and randomly scattered throughout the pellet. Macroscopic collapsed pellets can be found in Batch 2 of the non-annealed pellets, as some of these pellets have visually deformed structures with bubbles, cavities, and a shinier surface (Fig. 1).

The annealed pellets had a coarser structure and thicker materials compared to the non-annealed pellets. The structure has a sheet-like arrangement, where the sheets are oriented towards the centre of the pellet. There is no visual macroscopic collapse amongst the annealed samples, but small regions of microscopic collapse can be detected. The different annealing methods gave similar overall structures, but there are however minor differences. The pellets annealed for 1 week at -20 °C have notably thicker material and smoother structure compared to pellets annealed for 2 h at -9 °C. The representative material thickness estimated from the SEM images is > 1 µm for pellets annealed for 2 h at -9 °C and > 2 µm for pellets annealed for 1 week at -20 °C.

#### X-ray Microtomography

**Fig. 4:**
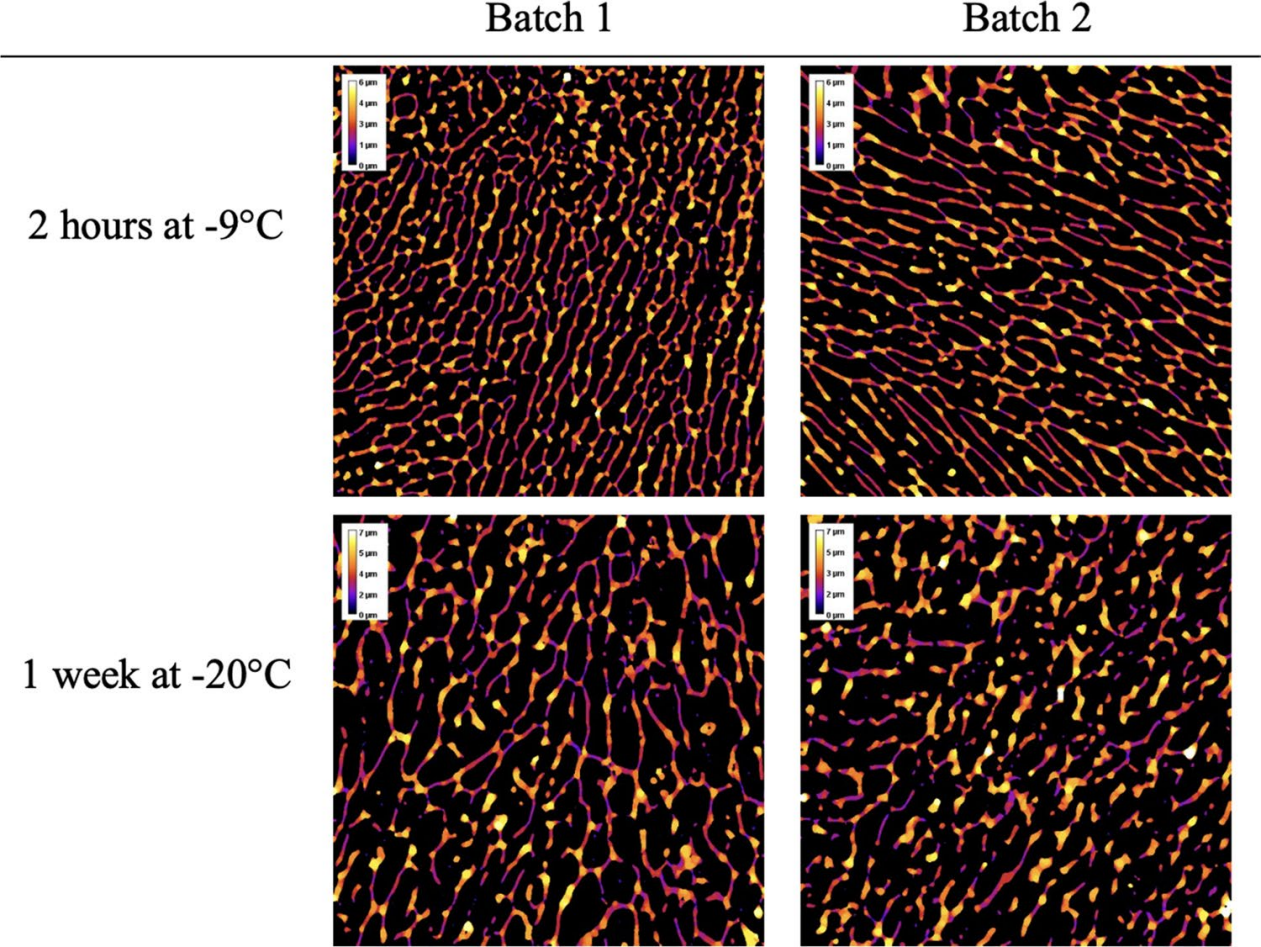
µCT slices of the color-coded local thickness variation, as quantified in 3D. Annealed samples at 2 h, -9 °C respectively at 1 week, -20 °C were investigated with tomography after drying

**Fig. 5:**
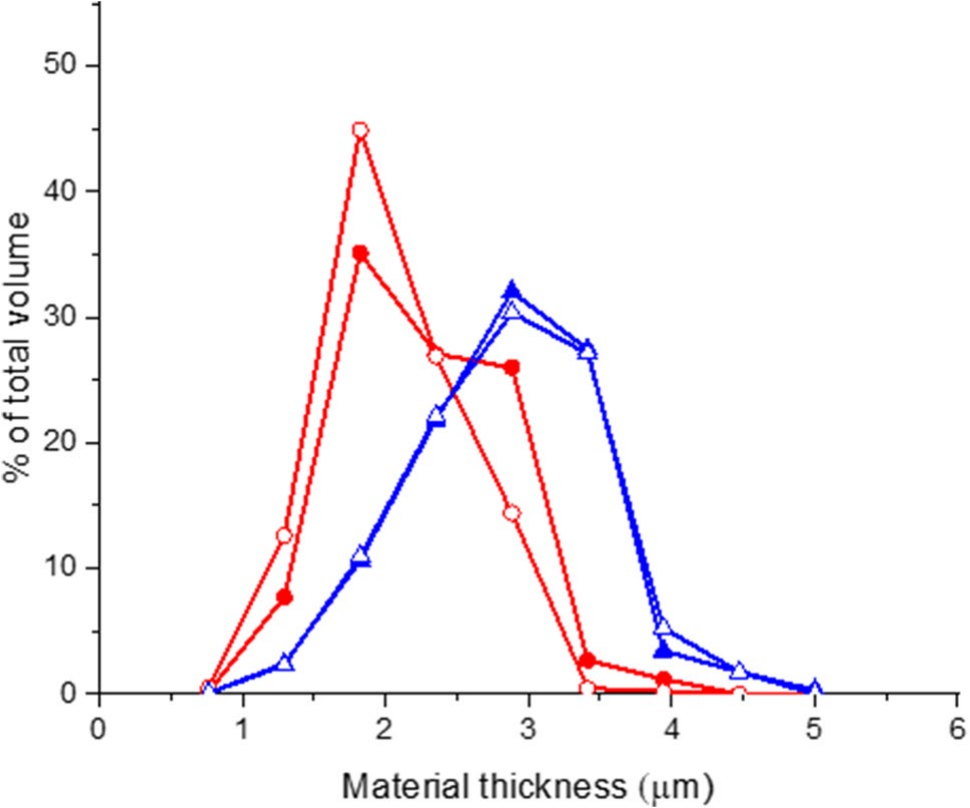
The volume-weighted material thickness distribution was obtained via image analysis of the µCT data sets of the samples, (Batch 1 ○, Batch 2 ●) annealed for 2 h at -9 °C, and (Batch 1 ▲, Batch 2∆) annealed for 1 week at -20 °C

Due to limits in resolution, it was not possible to reconstruct a representative image of the fine and dense structure of the non-annealed pellet. Thus, only the annealed samples were investigated. The results are presented in Fig. 4. It can be noted that the tomographic image reveals the same anisotropic pore character as the SEM image. The most important information acquired from tomography is the quantification of the material thickness, which was quantified in the 3D image using virtual spheres, ‘blobs’ embedded in the material. The analyses provided a number distribution of blobs that was converted to a volume-weighted distribution. A volume-weighted distribution is a more representative way to analyze the embedding capacity of the freeze-dried material (7, 8). The results show that samples annealed for 1 week at -20 °C had a mode value of 3 µm and a higher ratio of material thicker than 2.5 µm compared to samples annealed for 2 h at -9 °C which had a mode value of 1.5–2 µm, the results are shown in Fig. 5.

In the previous investigation (8), a similar quantification was made using a formulation consisting of maltodextrin where 1 h respective 4 h annealing at -7 °C gave 2.0 µm respectively 2.4 µm in material thickness. Thus, the 3 µm thick structures obtained here after 1 week at -20 °C can be considered a rather thick structure.

## Discussion

In this study, the structure of differently annealed pellets has been investigated using SEM and µCT to correlate the storage stability of a probiotic bacteria with the encapsulating material thickness. When evaluating the SEM images and µCT data, it is evident that non-annealed pellets have the thinnest material (below the tomographic resolution) and the pellets annealed at -20 ˚C for one week have the thickest material. The other major difference is the appearance of collapsed areas, where the annealed samples show little to no signs of microscopic collapse in the SEM and µCT images, while the non-annealed samples had extensive microscopic collapse and even macroscopic collapse. The non-annealed pellets in Batch 2 had the greatest extent of collapse where large cavities and dents were visually evident. Unfortunately, only intact pellets without visual signs of collapse were investigated using SEM or µCT, but based on previous experience (8) and other studies (11), a visual collapse leads to much thicker material structures.

**Fig. 6:**
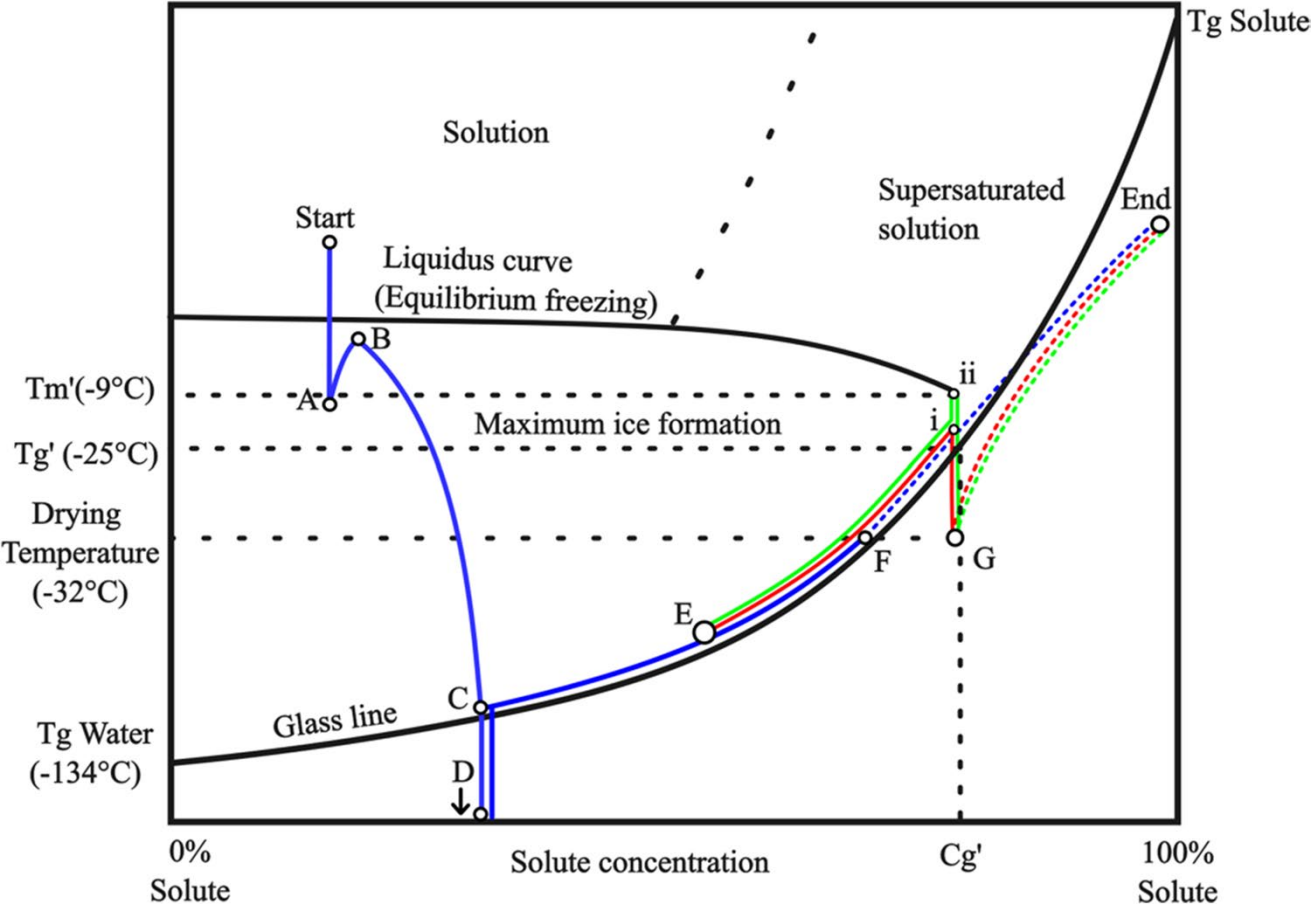
State diagram depicting the formulation comprising of 1/3 maltodextrin (DE 9) and 2/3 sucrose, has its Tg' experimentally measured, with other aspects assumed based on similarities to other systems. Trajectories in blue solid line represent the freezing and storage step for all pellets. A signify the nucleation of undercooled formulation, creating a two phase system comprising of ice crystals and freeze concentrate. The nucleation increases the temperature of the freeze concentrate (**B**), followed by rapid cooling and crystalization of numerous small ice crystals, further concentrating the freeze concentrate (**C**). Beyond point (**C**) the freeze concentrate is quenched and further crystalization is stopped (**C**-**D**). Upon storage, the temperature is raised to -80 °C, and water continues to diffuse from the freeze-concentrate to the ice crystals (termed relaxation) until the glass transition concentration at -80 °C is reached (**E**). The trajectories of the annealing process are signified by a solid red line for pellets annealed at –9 °C (i) and a solid green line annealed at -20 °C (ii). Annealing results in a freeze concentrate composition corresponding to Cg’. The freeze-drying occurs at -32°C and is represented by dotted lines. This can contribute to further relaxation for the non-annealed pellets to point (**F**), while the starting point of drying for the annealed pellets starts at point (**G**). Notice the difference in the drying starting position between annealed(**G**) and non-annealed pellets (**F**). After the ice has been evaporated, the sample is further dried to the stable End point

The emergence of structures and collapses is dependent on the freezing, relaxation, and annealing procedures (8). The trajectory of these steps in a state diagram can be visualized in Fig. 6. Due to the rapid freezing, a large number of very small ice crystals are formed, which grow as water diffuses from the freeze-concentrate to the ice crystals. The rapid cooling also results in non-equilibrium freezing where the freeze-concentrate drops below Tg before reaching a maximally freeze-concentrated solution, resulting in a freeze-concentrate with lower Tg than Tg’. When quenched pellets reside in temperatures above their Tg, water from the freeze-concentrate can migrate to the ice crystals. This increases the size and the total volume of ice crystals, further concentrates the freeze-concentrate, and increases its Tg. The relaxation process is rapid and occurs when the pellets reside inside the -30 °C pre-cooled freeze-dryer. The rapid rate of relaxation makes it a difficult process to avoid in practice. Annealing refers to the process of ice crystal growth driven by Ostwald ripening and occurs at temperatures above the Tg’ of the system. In this process, water is transferred from the small crystals to the larger crystals, resulting in larger more even-sized ice crystals. The annealing process does not increase the overall volume of ice crystals and should not be confused with relaxation where the water is transferred from freeze-concentrate to the ice crystals.

Quenched non-relaxed pellet possesses small ice crystals and a high proportion of freeze concentrate with a low Tg, resulting in an unstable material prone to.

global and extensive collapse during drying (8). This is evident in Batch 2 of the non-annealed pellets. While Batch 1 had sufficient time inside the -30˚C pre-cooled freeze-dryer to relax and become more stable. Collapse may also be induced by high mass transport resistance for a material consisting of a very fine structure and small pores. The high resistance leads to an increase in pressure at the sublimation front, this may lead to a temperature increase above Tg that causes movements in the material. Thus, collapse is more likely to be observed for the non-annealed pellets than for the annealed ones.

**Fig. 7:**
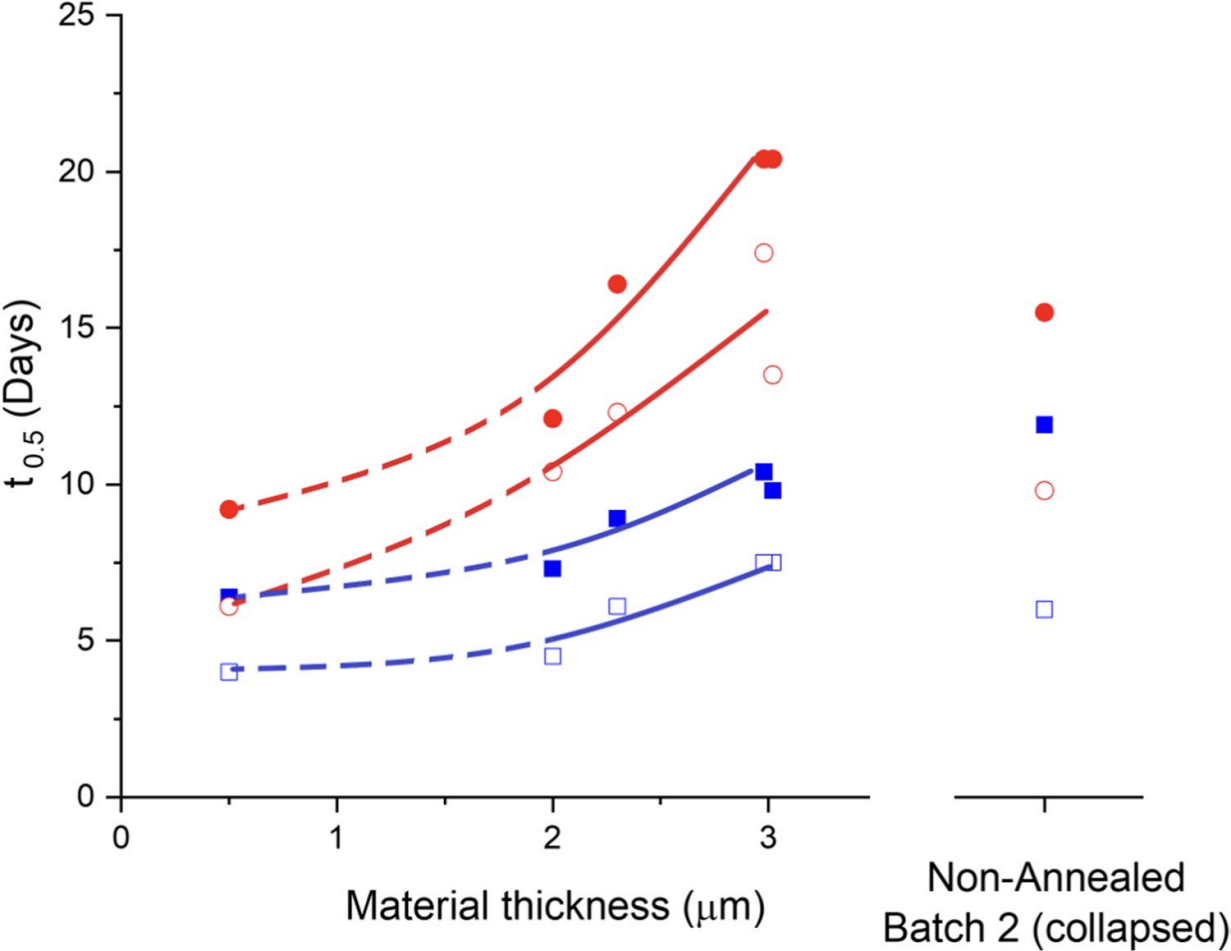
Stability of pellet freeze-dried Limosilactobacillus reuteri as a function of material thickness. The stability is observed as the half-time period of the viability (Eq. 1–3) measured during storage at 37°C. Half-time period for (1 month □, 2 months ■) Stored in canister, (1 month ○, 2 months ●) stored in a vacuum bag. The dimension of the material thickness has been measured using µCT and quantified using image analysis for all samples above 1.5 µm. For samples with thinner material, the material thickness was estimated from the SEM images and is therefore not fully comparable with the tomographic datasets. The non-annealed sample from Batch 2 had a macroscopically heterogeneous structure that could not be quantified in a meaningful way and is thus presented individually at the right panel in the figure

The loss of viability can in many cases be described by an exponential decay (21) (Eq. 1). This aligns reasonably well with the information presented in Fig. 2, although with a more rapid initial loss of viability. The slope can be described by the half-life period of the viability (*t*_*1/2*_, Eq. 3). The *t*_*1/2*_ values in Table III are compared with the material thickness and presented in Fig. 7. The results show a strong correlation between increasing material thickness and increased storage stability. Where the most annealed and collapsed pellets (thickest material) yielded the highest stability.

The deviation in stability between the batches annealed at -9 °C, 2 h is considerably larger than the deviation between the batches annealed at -20 °C, 1 week. The deviation is also evident when investigating the material thickness where Batch 1 has a slightly higher proportion of thicker material compared to Batch 2. The stability is therefore also higher for Batch 1. The reason for the deviation is unclear but can be due to a small difference in residence time during annealing. This aspect warrants further investigation. The large deviation in appearance and storage stability between the non-annealed pellets in Batch 1 and Batch 2 is likely due to the difference in degree of relaxation. Batch 1 resided inside the -30˚C freeze dryer for 10–15 min longer compared to Batch 2. This is enough time for Batch 1 to become slightly relaxed, which would lead to improved mass transfer conditions (11, 22). As a result, the short annealed (Batch 1) pellets maintain their microstructure while non-annealed (Batch 2) results in large collapse and a much thicker encapsulating material. The influence of a short relaxation (5–30 min) on the material thickness of quenched pellets is also evident in the work by Ekdawi-Sever et al. The study further revealed that collapsed pellets featuring thicker material exhibited increased storage stability, this correlation was linked to water content rather than the material thickness (where water content showed a covariance with material thickness)(8, 11). In his study, the water content varied from 1–8%, while the variation in water content in this study was low, between 3.5–4%. Therefore, the water content is considered to have a low impact on the storage stability in this study.

Apart from the impact of material thickness the results also show that the samples stored in vacuum-sealed aluminium bags have significantly higher stability than the samples stored in canisters with desiccant. A possible factor contributing to the increase in stability with increasing material thickness may be correlated to the protection against oxygen in the freeze-dried material. The encapsulating material is composed of amorphous sucrose and maltodextrin which are hydrophilic and should therefore result in slow diffusion of oxygen which is more hydrophobic. Hence, an increased thickness of the encapsulating material will act as a more efficient barrier against oxygen. Kurtmann et al. observed a significant instability of freeze-dried *Lactobacillus acidophilus* under an oxygen-rich atmosphere, while it remained stable under an oxygen-depleted atmosphere. The bacteria were also more stable when there was an antioxidant present in the formulation. Kurtmann et al. also showed that a high water activity (> 0.1) can be detrimental to the storage stability (23). As the water activity remained low for all samples during storage, the water activity is considered to have a limited influence on storage stability.

## Conclusion

This paper shows the importance of the material thickness of freeze-dried pellets on the stability of the encapsulated bacteria. Primarily, the storage stability showed a strong correlation with the material thickness. Further, the study shows the negative effect of oxygen on storage stability and indicates that thicker materials made of amorphous carbohydrates might hinder the transport of oxygen.

A range of material thicknesses can be obtained by using different annealing protocols and more extensive annealing results in a thicker material. Different degrees of relaxation between batches for the non-annealed pellets resulted in large deviations in structure and storage stability. The deviation between batches was shown to decreased with increasing annealing, where annealing at -20 °C for one week resulted in a more consistent structure, compared to annealing at -9 °C for two hours.

The benefits of applying extensive annealing are therefore both to increase the storage stability of the probiotic product and to gain control of the freeze-drying process to ensure a homogenous and reproducible product.

### Supplementary Information

Below is the link to the electronic supplementary material.

Supplementary file1 (DOCX 13 KB)

## Data Availability

Detailed data can be sent upon request.
